# Violent crime datasets: Incidence and patterns in Malaysia from 2006 to 2017

**DOI:** 10.1016/j.dib.2019.104449

**Published:** 2019-09-03

**Authors:** Hashom Mohd Hakim, Hussein Omar Khan, Hafezul Helmi Hamzah, Mohammad Faiz Othman, Bryan Raveen Nelson, Geoffrey Keith Chambers, Hisham Atan Edinur, Mohd Tajuddin Abdullah, Nur Syahmina Rasudin

**Affiliations:** a DNA Databank Division (D13), Criminal Investigation Department, Royal Malaysia Police, 50560, Bukit Aman, Kuala Lumpur, Malaysia; b School of Industrial Technologies, Universiti Sains Malaysia, 11800, Pulau Pinang, Malaysia; c Intelligence/Operation/Record Division (D4), Criminal Investigation Department, Royal Malaysia Police, 50560, Bukit Aman, Kuala Lumpur, Malaysia; d Institute of Tropical Biodiversity and Sustainable Development, Universiti Malaysia Terengganu, 21030, Kuala Nerus, Terengganu, Malaysia; e School of Biological Sciences, Victoria University of Wellington, P.O. Box 600, Wellington, 6140, New Zealand; f School of Health Sciences, Universiti Sains Malaysia, Health Campus, 16150, Kubang Kerian, Kelantan, Malaysia; g Environmental Futures Research Institute, Griffith University, Nathan, Queensland, 4111, Australia; h School of Marine and Environmental Sciences, Universiti Malaysia Terengganu, 21030, Kuala Nerus, Terengganu, Malaysia

**Keywords:** Crime incidence, Crime pattern, Malaysia, Violent crime

## Abstract

This article provides violent crime data in Malaysia from 2006 to 2017. The violent crimes include murder, rape, gang robbery, robbery and voluntarily causing hurt cases. A total of 330,395 violent crime cases were reported in this 12 year period and the data were tabulated state by state for all thirteen states of Malaysia, including two states in Borneo (Sabah and Sarawak) and one federal territory (Kuala Lumpur). In general, violent crimes show a decreasing trend from 2006 to 2017 in Malaysia. However, armed gang robbery and armed robbery show a fluctuating pattern from 2008 to 2011. A similar pattern was also recorded for unarmed gang robbery from 2008 to 2010. The violent crime data deposited here are available for further analysis, e.g., for identifying risk factors such as demography, lifestyle, socio-economic status, government policies etc. which may be associated with violent crime incidence and pattern across the country.

**Table:** Specification Table

Subject area	Social science
More specific subject area	Criminology
Type of data	Tables and figures
How data were acquired	Violent crime data were obtained from Police Reporting System (PRS) with written permission from Inspector General of Police, Malaysia. Data reported here were previously used to highlight the importance of DNA databanking in Malaysia [1] and were analysed using descriptive analysis implemented in a Statistical Package for the Social Science software (IBM SPSS, Version 25, Released 2017, Armonk, NY: IBM Corp).
Data format	Raw and analysed
Experimental factors	Violent crime data from 2006 to 2017.
Experimental features	Reported murder, rape, gang robbery (armed and unarmed), robbery (armed and unarmed) and voluntarily causing hurt cases state-wide by year
Data source location	Kuala Lumpur, Malaysia
Data accessibility	Data are within the article
Related research article	H.M. Hakim, J. Lalung, H.O. Khan, N.R. Khaw, S. Narayanen, G. K. Chambers, H.A. Edinur, Experiences, challenges and the future direction of forensic DNA data banking in Malaysia, JSSM, 14 (2) (2019) 127–141 [1]

**Table:** 

**Value of the data** • This article brings together for the first time violent crime data in Malaysia from 2006 to 2017 and are important source of information for readers to learn trends of violent crimes in the country. • Violent crime patterns highlighted here are highly relevant and valuable to law enforcement agencies and can be used by them as a reference to design and improve their crime prevention programmes • The violent crime datasets deposited here can further be examined by other researchers (e.g., by looking at crime rates and patterns in relation to socio-economic factors such as employment rate, lifestyle and gross domestic product)

## 1 Data

**Fig. 1:**
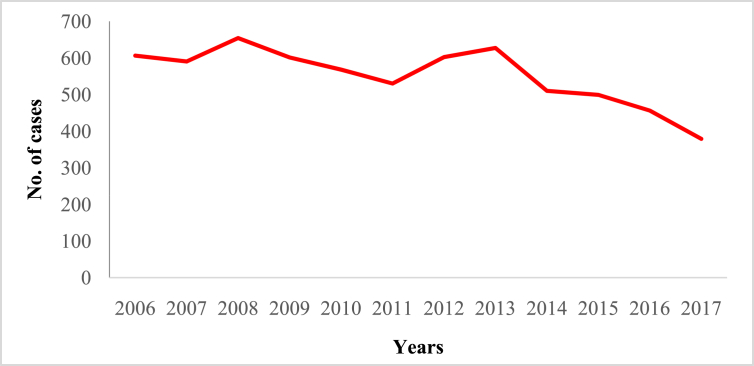
The pattern of murder cases for 12 years.

**Fig. 2:**
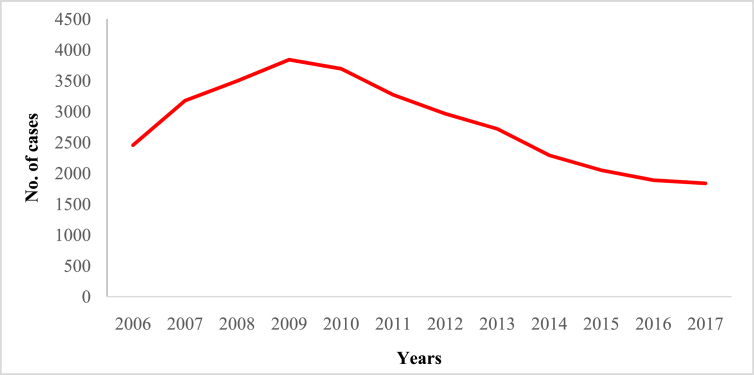
The pattern of rape cases for 12 years.

**Fig. 3:**
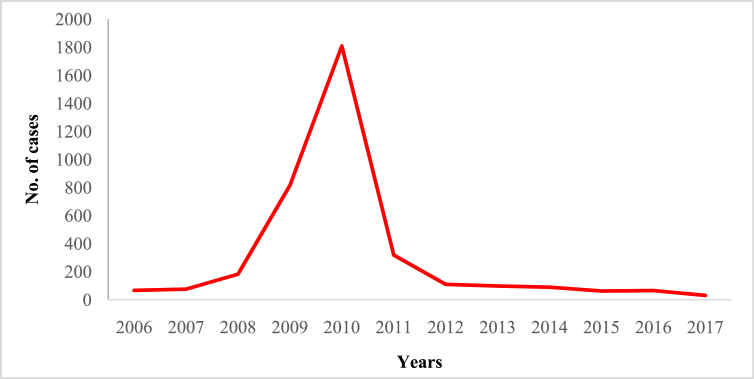
The pattern of armed gang robbery cases for 12 years.

**Fig. 4:**
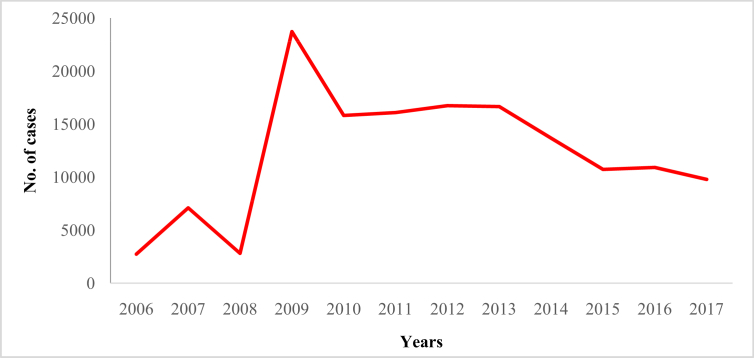
The pattern of unarmed gang robbery cases for 12 years.

**Fig. 5:**
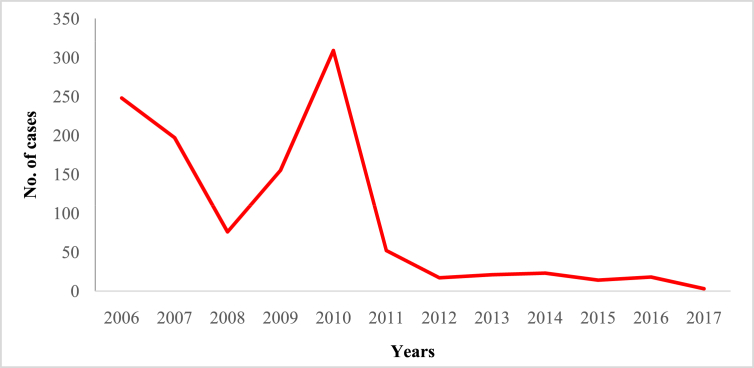
The pattern of armed robbery cases for 12 years.

**Fig. 6:**
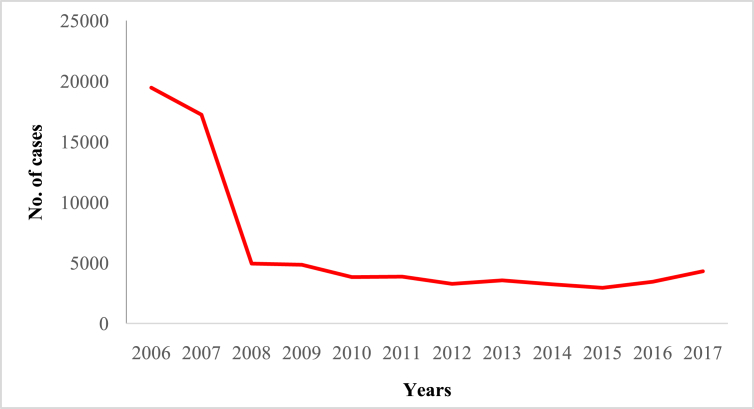
The pattern of unarmed robbery cases for 12 years.

**Fig. 7:**
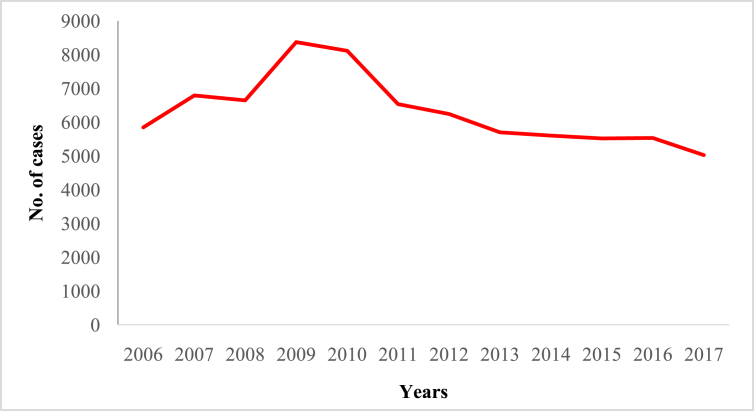
The pattern of voluntarily causing hurt cases for 12 years.

In this article we provide violent crime data (Table 1) in Malaysia from 2006 to 2017 (Table S1). These data were collected from thirteen states of Malaysia and one federal (Kuala Lumpur) territory (Tables S2–S15). The overall pattern of violent crime cases in Malaysia and by state are shown in Fig. 1, Fig. 2, Fig. 3, Fig. 4, Fig. 5, Fig. 6, Fig. 7 and Figs. S1–S14. Four categories of violent crime (murder, rape, unarmed robbery and voluntarily causing hurt) show a constant decreasing pattern from 2006 to 2017. However, a distinct elevation was observed for armed gang robbery and armed robbery for the period 2008–2011 and for unarmed gang robbery, 2008–2010. These due to the high numbers of cases reported in economically developed areas such as Selangor, Johor and Kuala Lumpur. In particular, Selangor was the highest contributor to armed gang robbery and armed robbery data, with 1048 and 152 cases respectively reported in 2010 (Figs. S15 and S16). Similar contributions also apply for unarmed gang robbery figures where 18,311 out of 23,722 cases were reported in Selangor, Kuala Lumpur and Johor in 2009 (Fig. S17).

**Table 1:** Definition of each type of violent crimes.

Type	Definition
Murder	Is defined and classified based on Section 300 Penal Code (Act 574), Laws of Malaysia: *Culpable homicide is murder*; *if the act by which the death is caused is done with the intention of causing death*; *if it is done with the intention of causing such bodily injury as the offender knows to be likely to cause the death of the person to whom the harm is caused*; *if it is done with the intention of causing bodily injury to any person*, *and the bodily injury intended to be inflicted is sufficient in the ordinary course of nature to cause death*; *or if the person committing the act knows that it is so imminently dangerous that it must in all probability cause death*, *or such bodily injury as is likely to cause death*, *and commits such act without any excuse for incurring the risk of causing death*, *or such injury as aforesaid*.
Rape	Is defined and classified based on Section 375 Penal Code (Act 574), Laws of Malaysia: *A man is said to commit “rape” who*, *except in the case hereinafter excepted*, *has sexual intercourse with a woman under circumstances falling under any of the following descriptions*; *against her will*; *without her consent*; *with her consent*, *when her consent has been obtained by putting her in fear of death or hurt to herself or any other person*, *or obtained under a misconception of fact and the man knows or has reason to believe that the consent was given in consequence of such misconception*; *with her consent*, *when the man knows that he is not her husband*, *and her consent is given because she believes that he is another man to whom she is or believes herself to be lawfully married or to whom she would consent*; *with her consent*, *when*, *at the time of giving such consent*, *she is unable to understand the nature and consequences of that to which she gives consent*; *with her consent*, *when the consent is obtained by using his position of authority over her or because of professional relationship or other relationship of trust in relation to her*; *with or without her consent*, *when she is under sixteen years of age*.
Robbery	Is defined and classified based on Section 390 Penal Code (Act 574), Laws of Malaysia: *In all robbery there is either theft or extortion*. *Theft is “robbery”*, *if*, *in order to commit theft*, *or in committing the theft*, *or in carrying away or attempting to carry away property obtained by the theft*, *the offender*, *for that end*, *voluntarily causes or attempts to cause to any person death*, *or hurt*, *or wrongful restraint*, *or fear of instant death*, *or of instant hurt*, *or of instant wrongful restraint*. *Extortion is “robbery”*, *if the offender at the time of committing the extortion*, *is in the presence of the person put in fear and commits the extortion by putting that person in fear of instant death*, *of instant hurt*, *or of instant wrongful restraint to that person or to some other person*, *and*, *by so putting in fear*, *induces the person so put in fear then and there to deliver up the thing extorted*. *The offender is said to be present if he is sufficiently near to put the other person in fear of instant death*, *of instant hurt*, *or of instant wrongful restraint*.
Gang robbery	Is defined and classified based on Section 391 Penal Code (Act 574), Laws of Malaysia: *When two or more persons conjointly commit or attempt to commit a robbery*, *or where the whole number of persons conjointly committing or attempting to commit a robbery*, *and of persons present and aiding such commission or attempt*, *amount to two or more*, *every person so committing*, *attempting*, *or aiding*.
Gang robbery/robbery (unarmed)	Is defined and classified based on Section 392-394 Penal Code (Act 574), Laws of Malaysia: *If any person*, *in committing or in attempting to commit robbery*, *voluntarily cause hurt*, *such person*, *and any other person jointly concerned in committing or attempting to commit such robbery’*.
Gang robbery/robbery (armed)	Is defined and classified based on Section 391 Penal Code (Act 574), Laws of Malaysia: *If at the time of committing or attempting to commit robbery*, *the offender is armed with or uses any deadly weapon*, *or causes grievous hurt to any person*, *or attempts to cause death or grievous hurt to any person*.
Voluntarily causing hurt	Is defined and classified based on Section 320-338 Penal Code (Act 574), Laws of Malaysia: *Whoever does any act with the intention of thereby causing hurt to any person*, *or with the knowledge that he is likely thereby to cause hurt to any person*, *and does thereby cause hurt to any person*. *Whoever voluntarily causes hurt*, *if the hurt which he intends to cause or knows himself to be likely to cause is grievous hurt*, *and if the hurt which he causes is grievous hurt*. *Voluntarily causes hurt by means of any instrument for shooting*, *stabbing or cutting*, *or any instrument which*, *used as a weapon of offence*, *is likely to cause death*, *or by means of fire or any heated substance*, *or by mean of any poison or any corrosive substance*, *or by means of any explosive substance*, *or by means of any substance which it is deleterious to the human body to inhale*, *to swallow*, *or to receive into the blood*, *or by means of any animal*.

## 2 Experimental design, materials, and methods

Violent crime data were obtained from the Malaysian Police Reporting System (PRS), which is used to record and store police reports from the public. The PRS was developed by Royal Malaysia Police in 2006 and can only be accessed by an authorized police officer. Each reported case is classified by an investigating officer for violent and property crimes and the PRS is available at district police stations, their contingent police headquarters (state level) and the RMP headquarters in Bukit Aman, Kuala Lumpur.

In Malaysia, only thirteen crimes are included in the national crime indices and seven (i.e. murder, rape, armed and unarmed gang robbery, armed and unarmed robbery and voluntarily causing hurt) of them are classified as violent crimes [2]. In this report, the violence crime data were extracted and tabulated from reported cases in the thirteen states of Malaysia and federal territory (i.e. Kuala Lumpur). We would like to note that data for Kuala Lumpur also include those violent crime cases recorded in Putrajaya territory, while data for Labuan territory was included with the Sabah records. The datasets were then properly compiled and analysed using descriptive statistics implemented in the Statistical Package for the Social Science software (IBM SPSS, Version 25, Released 2017, Armonk, NY: IBM Corp) and presented in the form of tables and graphical images.
