# Supplementary material for: Violent crime datasets: Incidence and patterns in Malaysia from 2006 to 2017
Source: Data Brief. 2019 Sep 3;26:104449. doi: 10.1016/j.dib.2019.104449 (PMC6811922; doi:10.1016/j.dib.2019.104449)
Supplement: Multimedia component 2 [file mmc2.doc]

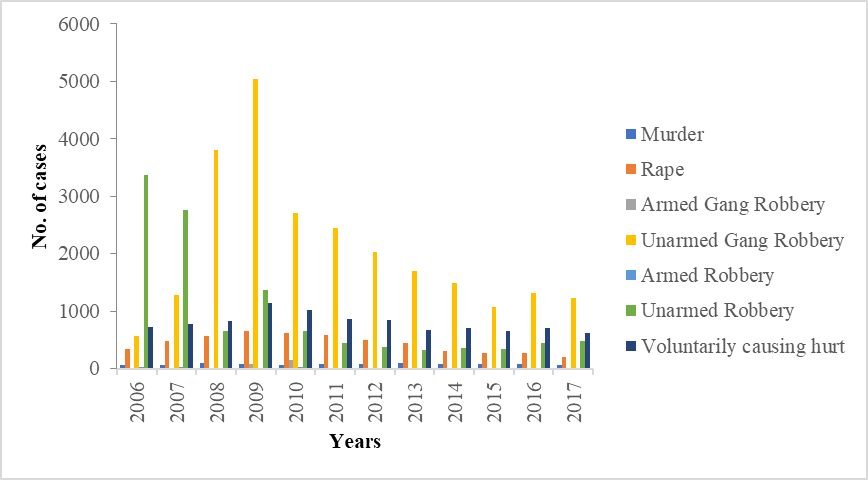


Fig. S1. Pattern of violent crime in Johor from 2006-2017


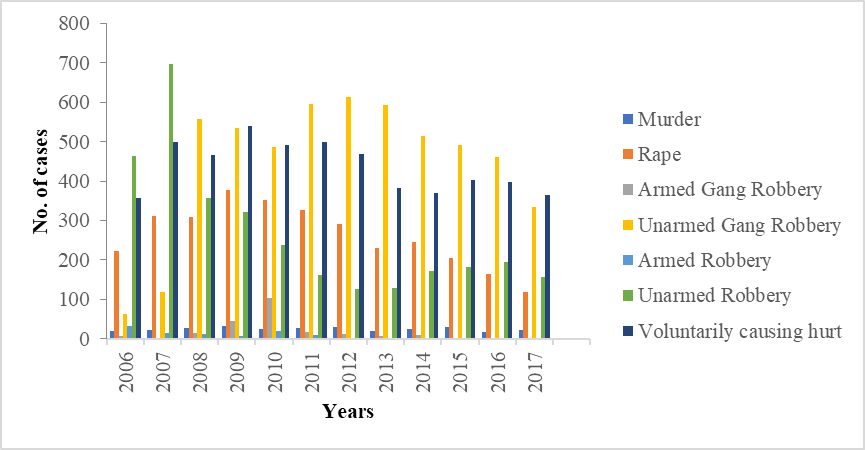


Fig. S2. Pattern of violent crime in Kedah from 2006-2017


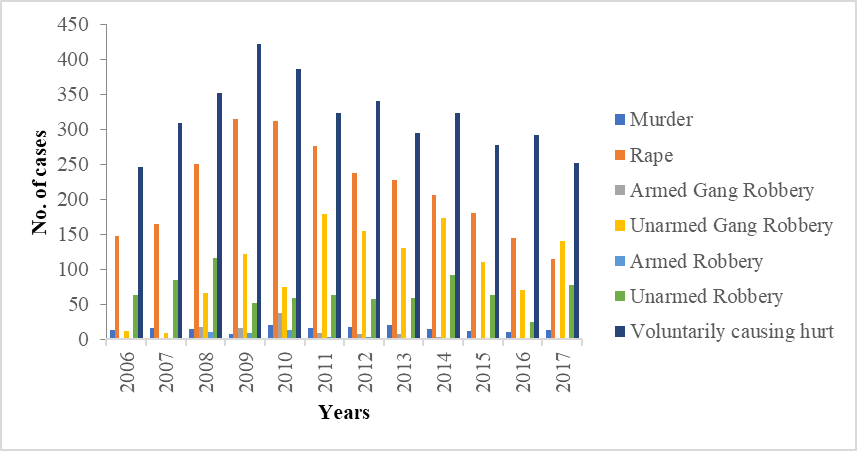


Fig. S3. Pattern of violent crime in Kelantan from 2006-2017


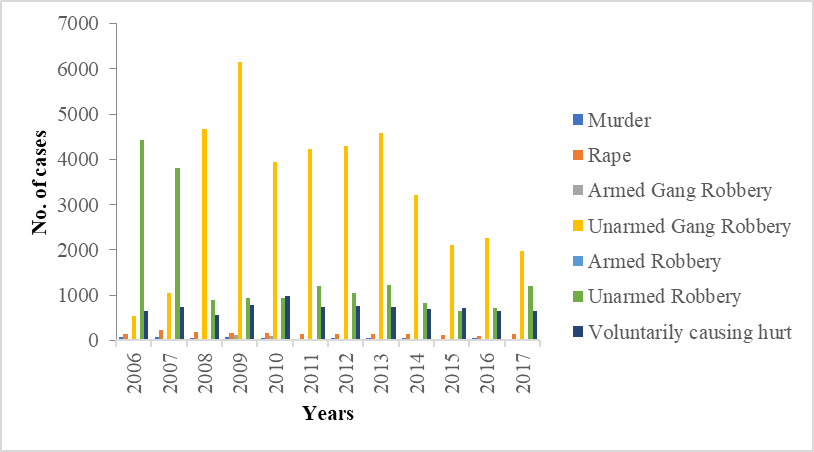


Fig. S4. Pattern of violent crime in Kuala Lumpur from 2006-2017


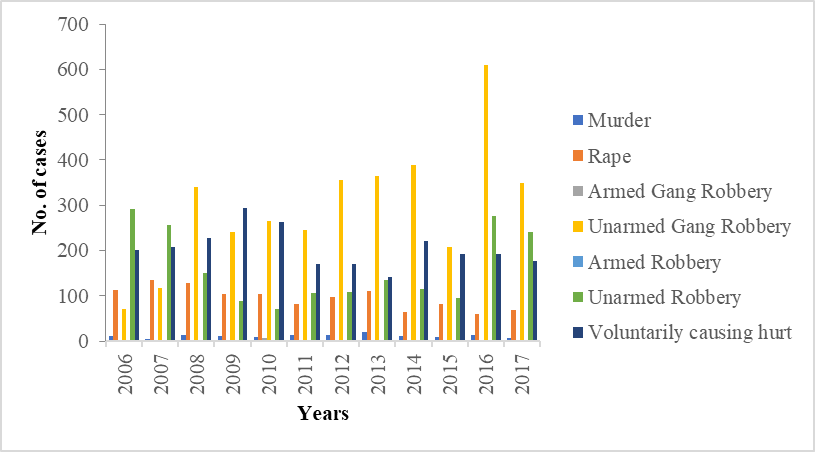


Fig. S5. Pattern of violent crime in Melaka from 2006-2017


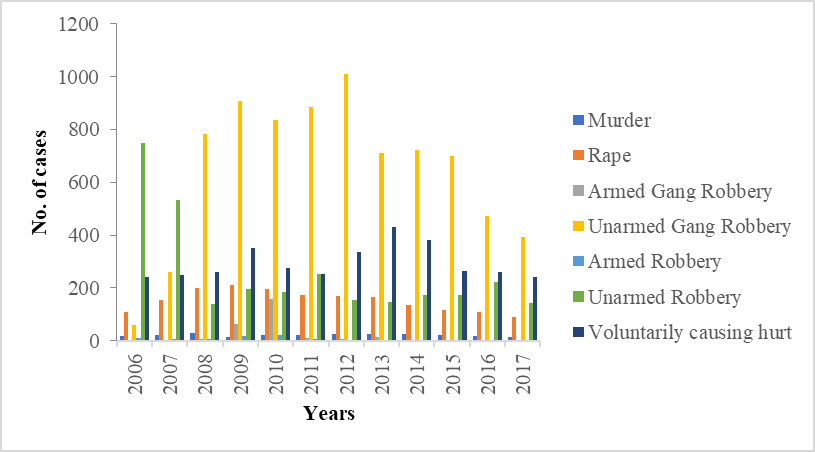


Fig. S6. Pattern of violent crime in Negeri Sembilan from 2006-2017


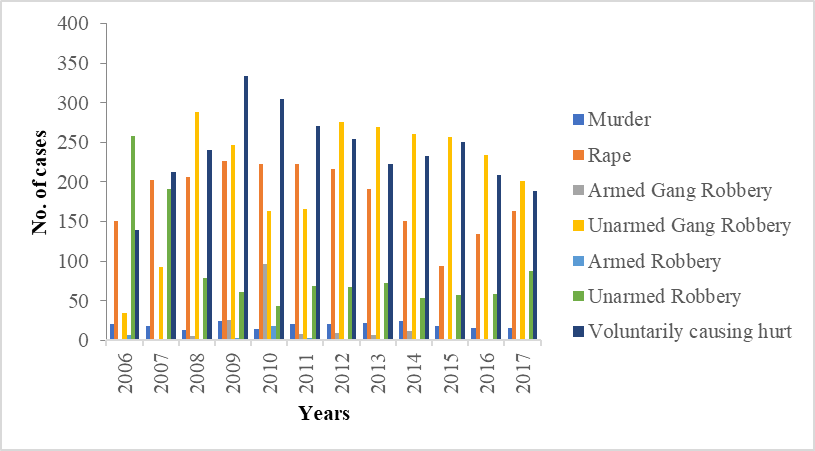


Fig. S7. Pattern of violent crime in Pahang from 2006-2017


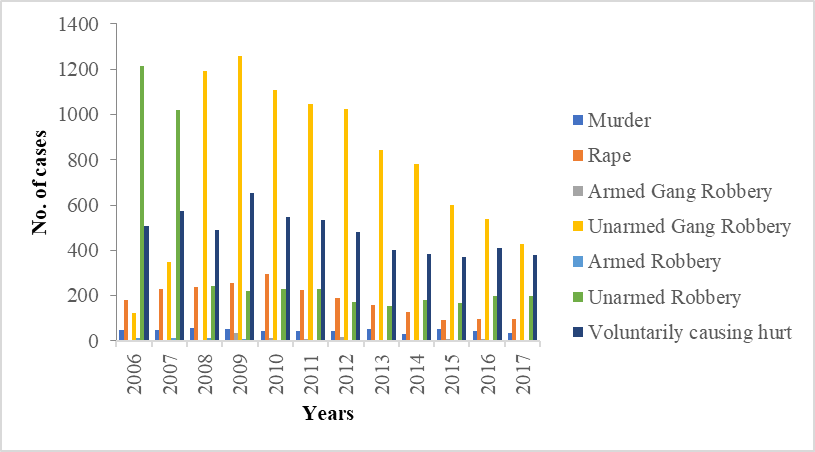


Fig. S8. Pattern of violent crime in Perak from 2006-2017


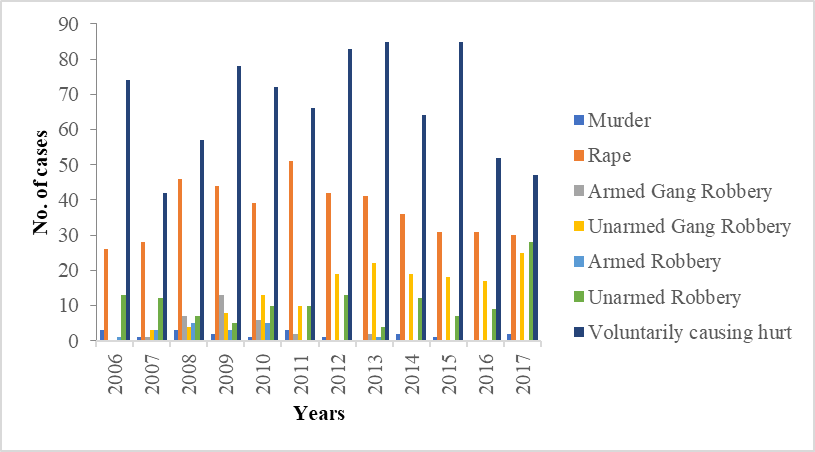


Fig. S9. Pattern of violent crime in Perlis from 2006-2017


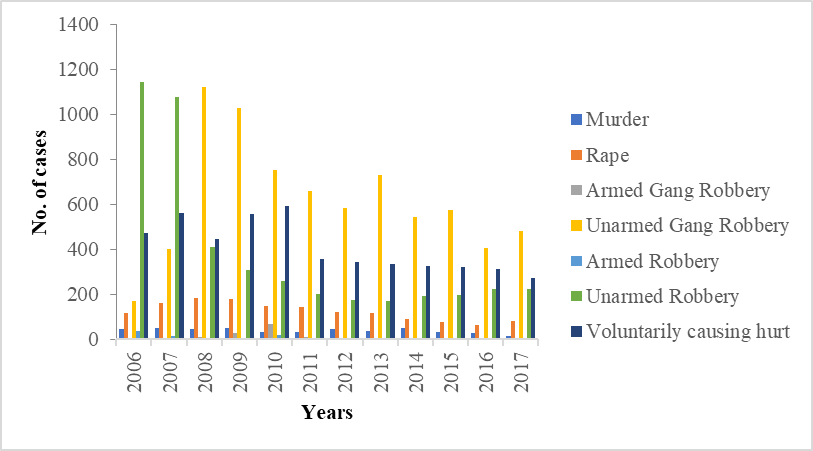


Fig. S10. Pattern of violent crime in Pulau Pinang from 2006-2017


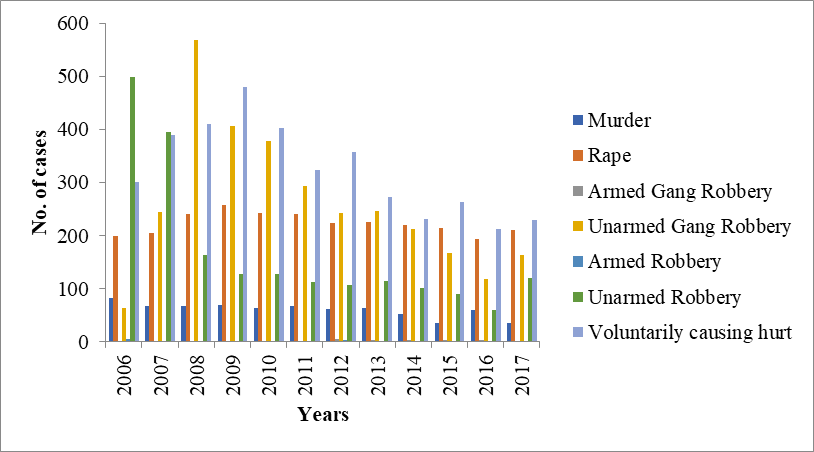


Fig. S11. Pattern of violent crime in Sabah from 2006-2017


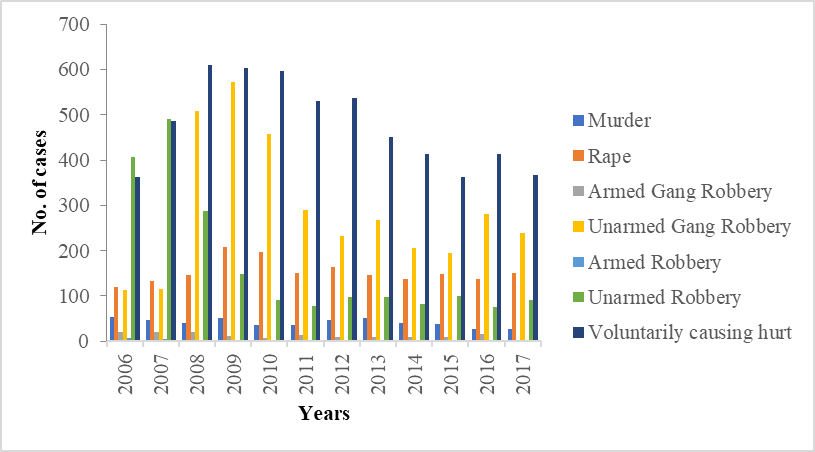


Fig. S12. Pattern of violent crime in Sarawak from 2006-2017


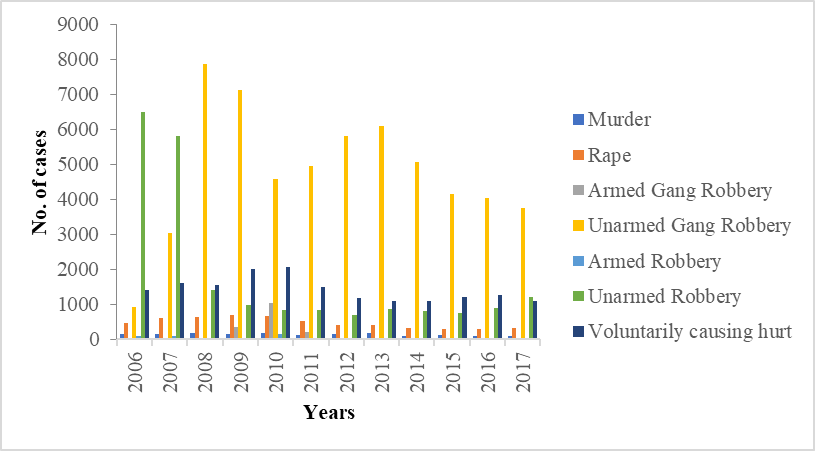


Fig. S13. Pattern of violent crime in Selangor from 2006-2017


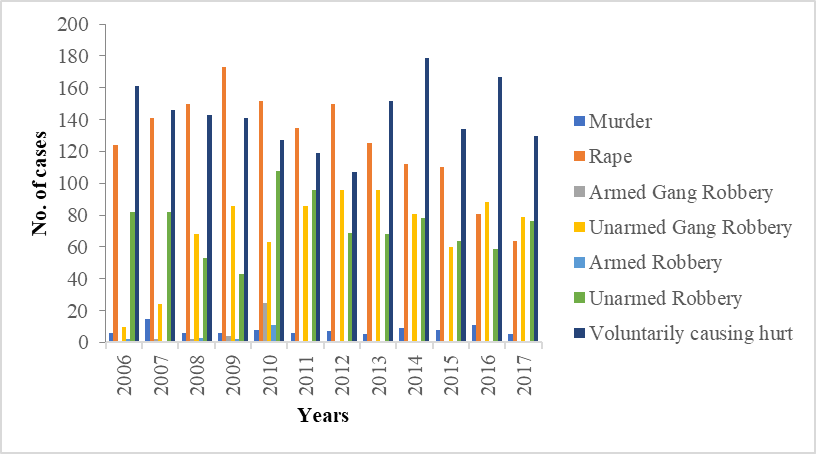


Fig. S14. Pattern of violent crime in Terengganu from 2006-2017


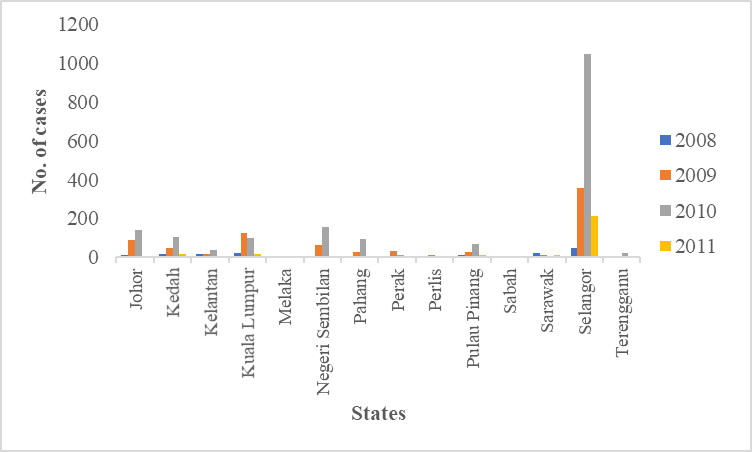


Fig. S15. Pattern of armed gang robbery from 2008-2011


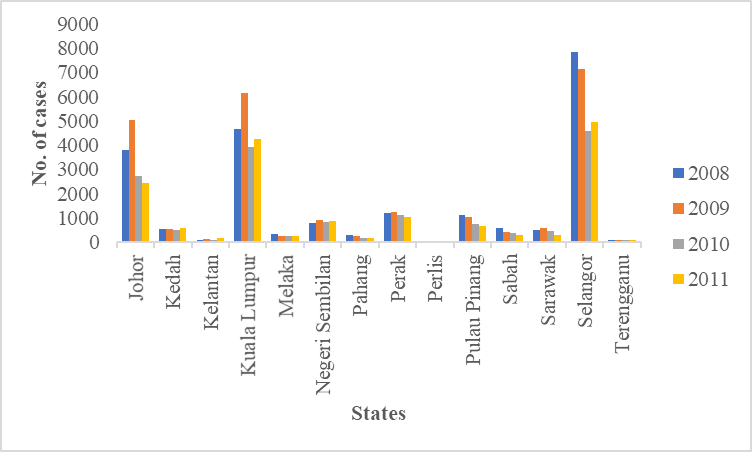


Fig. S16. Pattern of unarmed gang robbery from 2008-2011


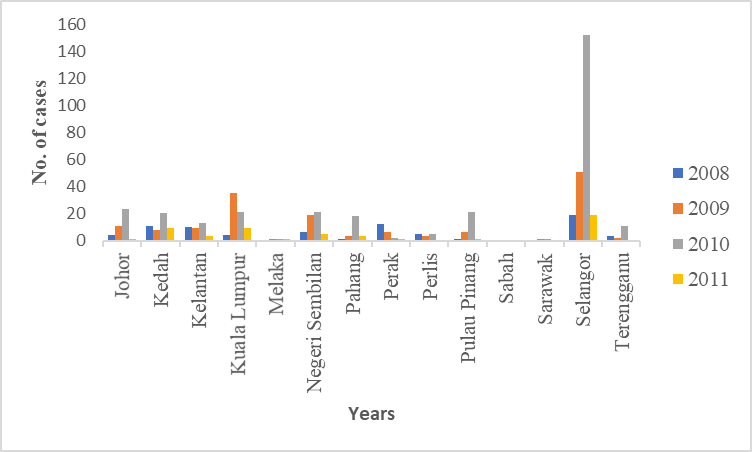


Fig. S17. Pattern of armed robbery from 2008-2011
